# Eosinophil Extracellular Traps and Inflammatory Pathologies—Untangling the Web!

**DOI:** 10.3389/fimmu.2018.02763

**Published:** 2018-11-26

**Authors:** Manali Mukherjee, Paige Lacy, Shigeharu Ueki

**Affiliations:** ^1^Department of Medicine, McMaster University and St Joseph's Healthcare, Hamilton, ON, Canada; ^2^Department of Medicine, Alberta Respiratory Centre, University of Alberta, Edmonton, AB, Canada; ^3^Department of General Internal Medicine and Clinical Laboratory Medicine, Akita University Graduate School of Medicine, Akita, Japan

**Keywords:** eosinophils, degranulation, extracellular traps, ETosis, cytolysis, airways, sputum

## Abstract

Eosinophils are an enigmatic white blood cell, whose immune functions are still under intense investigation. Classically, the eosinophil was considered to fulfill a protective role against parasitic infections, primarily large multicellular helminths. Although eosinophils are predominantly associated with parasite infections, evidence of a role for eosinophils in mediating immunity against bacterial, viral, and fungal infections has been recently reported. Among the mechanisms by which eosinophils are proposed to exert their protective effects is the production of DNA-based extracellular traps (ETs). Remarkably, DNA serves a role that extends beyond its biochemical function in encoding RNA and protein sequences; it is also a highly effective substance for entrapment of bacteria and other extracellular pathogens, and serves as valuable scaffolding for antimicrobial mediators such as granule proteins from immune cells. Extracellular trap formation from eosinophils appears to fulfill an important immune response against extracellular pathogens, although overproduction of traps is evident in pathologies. Here, we discuss the discovery and characterization of eosinophil extracellular traps (EETs) in response to a variety of stimuli, and suggest a role for these structures in the pathogenesis of disease as well as the establishment of autoimmunity in chronic, unresolved inflammation.

## Introduction

Eosinophils have intrigued physicians and scientists alike since the time of their first report in 1879. While lower numbers in systemic circulation (<0.5 × 10^9^/L) are generally considered healthy (1), increased peripheral numbers and, more importantly, tissue accumulation is associated with disease (2). Essentially, eosinophilia is best known in host responses to helminth infections, followed by the recognized pathological role of eosinophils as end-stage effector cells in allergic diseases such as asthma, atopic dermatitis, rhinitis, eczema, and related conditions. Eosinophils have also been implicated in non-allergic disease pathologies, such as Crohn's disease, chronic obstructive pulmonary disease (COPD), and more recently, non-atopic asthma. The common denominator underlying all these pathologies is the ability of eosinophils to secrete potent immunomodulatory factors stored as pre-formed mediators within their granules (cytokines, chemokines, growth factors), as well as *de novo* synthesized lipid mediators and oxidative metabolites (3, 4). Moreover, the presence of clusters of free intact, membrane-bound eosinophilic granules (FEGs) and cationic granule proteins [major basic protein (MBP), eosinophil cationic protein (ECP), eosinophil-derived neurotoxin, and eosinophil peroxidase (EPX)] have been identified in inflammatory foci in eosinophilic diseases (5, 6). Indeed, presence of FEGs and cationic granule proteins is a better marker of disease activity than intact eosinophils as reported for asthma (5, 7). Despite their association with diseased states, granule proteins have been naturally (evolutionarily) selected for host defense against viruses, bacteria, fungi, and helminths [reviewed elsewhere in (8)]. The mechanisms of mediator release and tissue dispersal of eosinophil granules have therefore remained a topic of intense research.

## Eosinophils: degranulation, primary lysis, and granule release

In response to receptor stimulation, eosinophils typically release their granules (intact) or products of granules through degranulation. Degranulation is an umbrella term used to define processes where there is release of granule proteins from viable cells, or the release of intact/ruptured granules from “dying” cells, without reference to the specific underlying regulatory mechanism (6, 9). Four modes of degranulation have been reported for eosinophils using transmission electron, confocal, and super resolution microscopy studies, namely: (i) *classical exocytosis* (ii) *compound exocytosis* (10); (iii) *piecemeal degranulation* (PMD); and (iv) *cytolysis* [reviewed extensively, (9)].

The fourth physiologic form of degranulation is *cytolysis* which involves chromatolysis (disintegration of the chromatin of cell nuclei), followed by rupture of the cell's plasma membrane, leading to release of FEGs (11). Research in the past decade has suggested that eosinophil cytolysis leading to the production of FEGs may be a major *modus operandi* of eosinophils *in vivo*, and is not a crush artifact of *in vitro* experimentation [release of cell granules due to mechanical damage to cells or inadequate tissue handling (12)]. Indeed, it is the second most commonly observed eosinophil degranulation mode after PMD in allergic tissues (13), ranging from 10 to 33% of all degranulation modes (13–15). Cytolysis has been observed as a dominant secretory mechanism in other diseases such as eosinophilic esophagitis (16). Cytolysis is also referred to as necrosis, where ruptured eosinophils release FEGs into the surrounding milieu (9). Whether cytolysis is synonymous to necrosis has been debatable ever since the early observations of FEG clusters in diseased inflammatory tissue. Persson and Erjefelt termed the phenomenon as “primary lysis” and concluded such events to be the fate of highly activated eosinophils (12). It is now considered that eosinophil cytolysis includes spilling of cellular contents, including nuclear materials such as histones and DNA, in addition to granular proteins onto the extracellular matrix, and can be from either live or lytic cells (17). The current review article focuses on recent observations of extracellular DNA traps associated with granule release from both live (18) and lytic eosinophils (19), largely defined as eosinophil “cytolysis,” and their role in host defense and potential contribution to disease pathology.

## Evolution of extracellular traps: ETosis

The defining feature of our immune system is host defense involving recognition and elimination of pathogens that endanger our health. Typically, innate and adaptive immunity work in a continuum for achieving immune homeostasis. In the innate circle, polymorphonuclear neutrophils (PMNs) have been well-studied as the first line of defense against invading pathogens, while eosinophils are classically known for their proliferation and recruitment in response to helminthic parasites. Phagocytosis of microbial pathogens by innate cells (neutrophils and macrophages) followed by intracellular lysosomal degradation is the best-known innate immune mechanism for host defense against these microorganisms.

While anti-microbial mechanisms associated with phagocytosis have been very well-characterized, an unexpected observation was recently made demonstrating that neutrophils were capable of releasing nuclear DNA onto extracellular pathogens to control their growth and proliferation. These extracellular DNA deposits were termed neutrophil extracellular traps (NETs), and were first proposed in 2004 as a phagocytosis-independent anti-microbial pathway (20). Neutrophil extracellular traps (NETs) were found studded with high local concentrations of anti-microbial agents (peptides, proteases, reactive oxygen species), released into extracellular matrix to ensnare or degrade virulence factors and aid in the killing of bacteria (20, 21).

Similar extracellular traps (ETs) have subsequently been observed from other cells of the innate family members, for example, mast cells (22), monocytes (23), tissue macrophages (24), and eosinophils (18, 25–27). Recently, even lymphocytes, key effector cells of the adaptive immune system, were demonstrated to eject ETs *in vitro* when incubated with serum obtained from systemic erythematous lupus (SLE) patients (28). Based on such elegant observations, the release of ETs from immune cells that was associated with rupture of the cell membrane, was considered to be a novel cell death pathway (distinct from necrosis and apoptosis), and referred collectively as ETosis [extensively reviewed in (29)].

ETosis is proposed to be an immune-protective host defense mechanism, particularly at barrier sites, that has been conserved through evolution. Indeed, ET-like structures containing DNA have been discovered in plant root-tips that protect against fungal infections (30). Regulated release of chromatin ETs that ensnares microorganisms has also been demonstrated in several invertebrates (remarkably, an acoelomate), and essentially strengthens the notion that DNA traps are evolutionarily conserved as a defense weapon (31).

Notably, eosinophil-derived ETs have been observed in higher vertebrate forms. For example, both neutrophils and eosinophils undergo ETosis to trap and kill *Haemonchus contorta*, a gastrointestinal nematode in ruminant animals. Being trapped in ETs, the migration of the larvae to sites of infection is limited (32). In the case of the bovine respiratory pathogen *Manheimia haemolytica*, ETosis from innate immune cells including macrophages was shown to limit the spread of this bacterium (33).

## Eosinophil extracellular traps: are eosinophils dead or alive?

**Figure 1:**
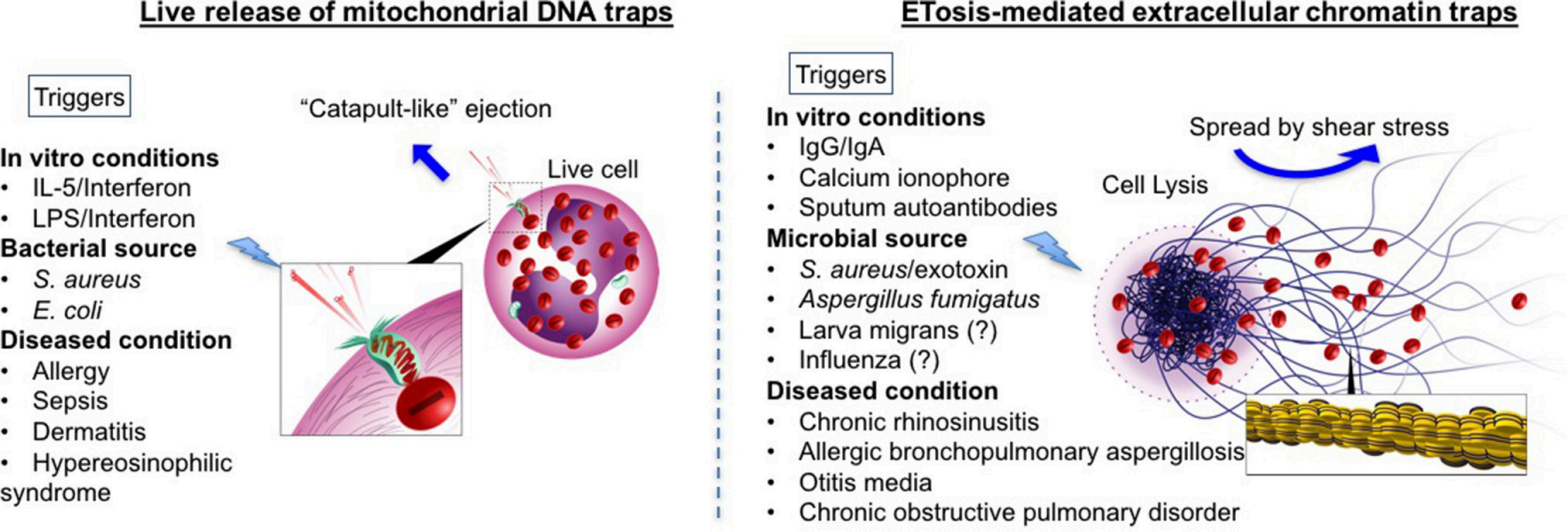
Comprehensive schematic of two theories of eosinophil extracellular trap formations: Comparison between mitochondrial DNA (mtDNA) traps **(Left)** and ETosis mediated extracellular traps **(Right)** are given. Blue arrows indicate how traps are spread extracellularly. In mtDNA theory, live eosinophil rapidly ejects mtDNA, which are loaded with specific eosinophil granule protein. MtDNAs are launched and spread into the extracellular space with a speed of at least 10–20 micrometer s^−1^, by unknown mechanism [suggested to be the stored elastic energy like plants catapulting pollen in the air (18)]. In contrast, ETosis-generated extracellular traps are mediated through active cell death program. Nuclear and plasma member disintegration during an event of cytolysis, allow for the release of chromatin-based web-like extracellular traps (as depicted in the enlarged image in square). Extracellular traps spread by a passive process. Shear stress (for example, cough, respiration, fluid flow including Brownian motion) enable to distribute free granules (red) and extracellular traps. Other triggers such as immunoglobulins and microbes have also been shown to release histone-coated EETs with evidence of cell death (19, 36, 37). The different triggers known (or reported in literature) to release EETs via both mechanisms have been listed (please note that this is not an exclusive list). For those with inconclusive evidence (or with evidence only from mouse models) have been indicated with a question mark.

Upon appropriate stimulation, eosinophils release intracellular DNA to form web-like ETs, embedded with granular proteins (refer to Figure 1). The deposition of extracellular DNA by immune cells is considered to be a double-edged sword, i.e., implicated both as a host defense machinery and in the development of certain pathologies. Compared to NETosis, coined by Steinberg and Grinstein, which is now accepted as a novel cell death pathway (21), the concept of extracellular trap formation with respect to eosinophils is conflicted. At present, there are two schools of thought regarding the formation of eosinophil extracellular traps (EETs) and the fate of cells releasing them (34, 35). In 2008, DNA net-like structures, similar to those reported for neutrophils, were observed for the first time by Yousefi et al. to be catapulted out of “live” eosinophils (18). Interestingly, the DNA present in these EETs was reported to be of *mitochondrial* origin, and DNA strands were embedded with granule proteins such as MBP and ECP, evident from co-localization studies both *in vitro* (18) and *in vivo* (27) (refer to Figure 1). Extracellular traps (ET) formation was independent of cellular cytoskeletal remodeling, since there was no inhibitory effect by cytochalasin D on EET release (18). The mitochondrial origin of EETs is yet to be confirmed in other reports (discussed in the next section).

In contrast to the above report, Ueki et al. (19) elegantly demonstrated the release of similar web-like structures from lytic eosinophils upon multiple triggers (demonstrated previously to cause eosinophil cytolysis), such as immobilized IgG, IgA (1 mg/mL, 120 min) (38), and the calcium ionophore A23187 (1 μM, 60 min) (39) (refer to Figure 1). These findings were obtained using transmission electron microscope and confocal (immunofluorescence) microscopy. Immunostaining with anti-histone antibody on non-permeabilized cells post-treatment confirmed that the DNA was *nuclear* in origin (histone-coated). To ascertain the difference between ETotic, apoptotic and necrotic eosinophils, anti-Fas activated eosinophils (undergoing apoptosis with high annexin V staining) and heat-inactivated eosinophils (with characteristic necrotic blebs) were compared with those undergoing ETosis. In ETotic eosinophils, surface annexin V staining was weak (indicative of low/no phosphatidylserine on the outer surface of the cell membrane, and suggesting these are not apoptotic) compared to Fas-activated cells. In addition, necrotic blebs were not observed. The casting of filamentous ETs was preceded by chromatolysis, involving rupture of both nuclear and cell membranes [events noted in primary lysis (11)].

Concurrently with EETs, intact membrane-bound granules were also observed to be released from cells. Since EET formation occurred in parallel with eosinophil lysis, the term EETosis was coined, and cells were presumed to undergo necrotic death (19, 35). Later, this was disputed, since EET formation does not always accompany cell death (40). Eosinophil extracellular traps (EETs) were shown to be released from live eosinophils as depicted by Yousefi et al. (18) and Gevaert et al. (41), and it is interesting that EETosis was dependent on NADPH (nicotinamide adenine dinucleotide phosphate) oxidase activity. In this study, EETs were studded with granule proteins, suggesting degranulation; properties that resemble EETotic eosinophils observed by Ueki et al. (19). It is also perhaps compelling to emphasize that the number of EET^+^ live eosinophils reported by Gevaert et al. (41) in nasal polyps (~8.8% of the total eosinophils) was similar to the percentage of lytic eosinophils with FEG clusters (9.9%) reported by Erjefält and co-workers in nasal tissue (13).

In recent years, there have been reports of EETs accompanied with features of cell lysis. Charcot-Leyden crystals, the classical feature of eosinophil “activity,” were shown to closely associate with EETosis both *in vivo* and *in vitro* (42). EETosis was demonstrated *in vivo* in induced sputum plugs from COPD (chronic obstructive pulmonary disease) patients. Increased eosinophil free granules and cellular debris were found associated with EETs, and MBP was shown to co-localize with observed DNA-based ETs (25). Again, immobilized IgG (50 μg/mL) and immunoprecipitated immunoglobulins from the sputum of severe eosinophilic asthmatics with increased anti-EPX IgG titers were shown to trigger extensive EETs *in vitro* from isolated peripheral eosinophils within 3–12 h of incubation (36). Sputum immunoglobulins with autoantibodies were more potent than affinity-purified IgGs from pooled healthy donors in inducing extensive histone-coated EETs in a time-dependent manner. Increased EETs over time was accompanied with an increase in extracellular lactose dehydrogenase (LDH) levels, indicative of membrane rupture and cell lysis (36).

According to a recent comment by Rosenberg and Foster (17), it is now realized that all EETs are not created equal, and some may be released from live cells while some emerge from lytic (necrotic) cells. However, the process of EET formation is morphologically and functionally distinct from any forms of programmed cell death. Looking at the current literature and ongoing discussion, it may be prudent to remark that the fate of eosinophils (whether dead or alive) during EET formation may be dependent on the stimuli either *in vitro* or *in vivo*. Specifically, *in vitro* experimental conditions such as priming, the nature of stimuli, the time-span involved, and the experimental technique used for assessment may have very distinct outcomes for EET formation from either live or dead cells. The incidence of live or dead eosinophils may also be dependent on the state of eosinophil activation itself at inflamed tissues/foci. For example, the same stimuli, such as *Staphylococcus aureus*, on isolated eosinophils *in vitro* caused EETs to catapult out of live cells within 15 min or less (37), while longer incubations with bacteria or other stimuli for >60 min allowed EET formation with concomitant FEG release (indicative of primary cytolysis) and reduced numbers of viable intact eosinophils (37). In contrast, LPS stimulation of IL-5/IFNγ-primed eosinophils with time-lapse automated confocal imaging confirmed that EETs from live eosinophils were released within 1–11 s, with the kinetics of DNA spread estimated at 10–20 μm s^−1^ (18). A later independent study demonstrated that EETs were released from activated eosinophils co-cultured with *S. aureus*/exotoxins (43), but the mechanism of EET release was not described.

## Source of eosinophil extracellular DNA traps: nuclear or mitochondrial?

There are several possible reasons that could explain the discrepancy in findings regarding the source of EETs being either mitochondrial or nuclear in origin. In the former study suggesting a mitochondrial source for EETs (18), the concern is that the ejection of small particles of mitochondrial DNA may be physically challenging due to high fluid resistance (“drag”) within the cytoplasm. The amount of energy required to eject mitochondria through the cytoplasm is predicted to be very high, and is akin to throwing strings from underwater so that they fly up into the air. In contrast, the formation of “DNA clouds” and “DNA traps” as reported elsewhere were typically observed as deriving from the nucleus of cells (19). Perhaps most importantly, human eosinophils contain very few mitochondria (24–36/cell compared with ~1,300/cell for hepatocytes), suggesting that very little mitochondrial DNA can be released (44). Each mitochondrion contains ~2–10 copies of mitochondrial DNA, which is highly susceptible to reactive oxygen species-mediated damage because it lacks protective histones and has limited DNA repair mechanisms. Finally, the lack of nuclear DNA in eosinophil supernatants may be explained by the highly adhesive properties of these DNA strands, which are likely to clump and adhere to cells and plate surfaces, particularly after shaking during incubation. This would precipitate the majority of nuclear DNA from supernatants while mitochondrial DNA, being smaller in its size, will remain soluble in the supernatant and more readily detected by PCR. In contrast, using sensitive molecular methods, increased release of dsDNA in the supernatant in an event of induced-EETs *in vitro* has been demonstrated (36). As described above, recent findings by Ueki et al. (19) suggest that most EETs consist of histone-bound DNA that is nuclear in its origins (26). Therefore, the ability of mitochondrial DNA to generate extensive and large DNA traps from eosinophils observed *in vitro* and *in vivo* is physically very challenging and conceptually difficult to apply. These discrepancies and subsequent speculations demand further investigation.

## EETs and host defense

As discussed in section Evolution of Extracellular Traps: ETosis, ET formation by innate cells is an evolutionarily conserved mechanism. Extracellular traps (ET) formation and function was first described in neutrophils, and these are well-known for their microbicidal properties both *in vitro* and *in vivo*, including mouse models of sepsis (45). In recent years, compelling evidence has accumulated suggesting similar roles for EETs and their possible role in host defense.

### EETs and parasites

Eosinophils are primarily implicated in helminthic parasite infections. ETosis has been proposed to be a novel extracellular killing mechanism for pathogens that are too large to allow phagocytosis. There is limited evidence regarding EETs and parasites. Although a recent veterinary report showed that both neutrophils and eosinophils release ETs to trap *H. contorta* larva, a known pathogenic nematode in ruminant animals (32), the role of EETs is doubtful due to the low purity of isolated eosinophils (30%) using Percoll gradients in this study. EET^+^ eosinophils have been reported in the deep dermis of skin biopsies from patients with parasitic infections (larva migrans, ectoparasitosis) (46) however, *in vitro* evidence of parasite-induced EETs has not yet been obtained.

### EETs and bacterial infection

As mentioned briefly in section Eosinophil Extracellular Traps: Are Eosinophils Dead or Alive?, primed eosinophils were demonstrated to cast EETs in response to a bacterial product trigger (LPS). Yousefi et al. (18) demonstrated that priming of eosinophils with IL-5 and/or IFNγ, or eotaxin, allowed catapult-like EET formation within 1–15 s on subsequent bacterial stimulation by *E.coli* (18) or *S. aureus* (41). Further, they demonstrated that activated eosinophils cultured with opsonized *E. coli* could kill approximately 90% of inoculated bacteria within 45 min by a phagocytosis-independent mechanism (18). The authors also demonstrated that in a model of post-caecal ligation and puncture, IL-5-transgenic and not wild-type mice showed demonstrable intestinal eosinophil infiltration and extracellular DNA deposition (indicative of EETs), which allowed protection against microbial sepsis (18). Prince et al. later demonstrated that EET release by lytic eosinophils, when co-cultured with *S. aureus*, was mediated by bacterial virulence factors (37). The first evidence of EETs embedded with eosinophilic granule proteins (ECP and MBP) *in vivo* was obtained in bacteria-infected caecum (*Spirochetosis*) (18). Caecal tissues in this model were infiltrated by EET^+^ eosinophils, rather than neutrophils (confirmed by the absence of a CD16^+^ population). It has also been demonstrated that EETs provide a larger adhesive surface that allows for encapsulation of fungi and bacteria (26).

### EETs and fungal infection

Eosinophils are prominent cells in allergic bronchopulmonary mycosis and fungal-associated asthma (47), but their molecular interactions and consequent immunopathological roles in fungal infections are yet to be conclusively defined. The first evidence of EETs forming in fungal disease was reported in a recent clinical case (48), where a patient diagnosed with ABPA (allergic bronchopulmonary aspergillosis) showed CT evidence of bronchiectasis and mucus plugging. Bronchial secretions from this patient exhibited an intense accumulation of eosinophils in the mucus and chromatolytic nuclei aggregated to form filamentous structures that co-stained with anti-human histone 1 antibody and Hoechst 33342 DNA stain. Clusters of free eosinophil granules attached to DNA traps were also detected (48), in agreement with their previous report that EETosis releases intact membrane-bound free eosinophil granules (19). In addition, using scanning electron microscope, the same group demonstrated the capture of *Candida albicans* by EETs *in vitro* (19). Recent evidence from Muniz et al. (49) revealed that *Aspergillus fumigatus* can induce EETs from isolated eosinophils *in vitro* in a ROS-independent manner, but occurred via CD11b binding (a receptor for fungal antigens) and activation of the Syk tyrosine kinase pathway (49). However, EETs were incapable of fungicidal activity. This is similar to findings relating to ETs released from neutrophils in response to *A. fumigatus*, where two independent studies failed to show any NET-related fungicidal activity (50), while there was some evidence that NETs could inhibit germination of fungal spores (51).

### EETs and virus infections

The role of eosinophil-induced ETs in limiting virus infections still remains to be elucidated. Interestingly, extracellular histones coated on EETs have recently been implicated in influenza-induced lung pathogenesis. Ashar et al. demonstrated a high accumulation of extracellular histones in mice infected with influenza virus that was associated with widespread pulmonary microvascular thrombosis (52). Increased accumulation of extracellular histones was also evident in nasal lavage from influenza-infected patients; however, this study did not investigate whether histones were generated due to EET formation. Neutrophil extracellular traps (NETs) were also observed to be induced by human respiratory syncytial virus *in vitro* (53), but there is no evidence of virus-induced EETs despite evidence of an anti-viral role for eosinophils (54). In fact, it is interesting that eosinophil recruitment to the lungs in response to *A. fumigatus* in a mouse model leads to active eosinophil degranulation and consequent protection from lethal respiratory virus infection (55).

## Extracellular traps in disease pathology

Despite their demonstrated and important role in host defense, ETs have also been associated with host damage, appearing in association with several pathologies including sepsis, diabetes, and autoimmunity (56). Most of our current knowledge about ETs is based on investigations of neutrophils and the phenomenon of NETosis [reviewed extensively (56)]. Neutrophil extracellular traps (NETs) are studded with self-antigens such as histones, dsDNA, and myeloperoxidase, all of which are implicated in a host of autoimmune diseases, and their role in autoimmune pathogenesis is well-defined (56–59). The association of free FEGs and eosinophil granule proteins with disease severity (5, 7, 15, 60), coupled with recent *ex vivo* evidence of EETs in diseased tissues (tabulated chronologically in Table 1) have led researchers to investigate and speculate possible contribution of EETs to disease etiology, sustenance, and progression in continuing studies of cell function, animal models, and clinical cases.

**Table 1:** *In vivo* evidence of eosinophil extracellular traps in human disease.

**Year**	**Disease**	**Sample**	**Methodology used**	**References**
2008	Crohn's disease	Paraffin-embedded intestinal sections	Confocal laser scanning microscope: anti-MBP polyclonal antibody, Sytox orange, and Mito tracker	(18)
2011	Asthma and allergic airways	Endobronchial biopsies	IF with propidium iodide and anti-MBP antibody	(27)
2011	Atopic dermatitis, allergic contact dermatitis, urticaria, bullous pemphigoid, hypereosinophilic syndrome, dermatitis herpetiformis	Skin biopsies	Confocal laser scanning microscopy with propidium iodide and anti-human ECP antibody	(46)
2013	Allergic sinusitis and hypereosinophilic syndrome	Tissue biopsies	Transmission electron microscopy	(19)
2015	Eosinophilic esophagitis	Esophageal biopsy	IF with propidium iodide and anti-human EPX antibody	(61)
2015	Eosinophilic cellulitis or Well's syndrome	Skin biopsies (flame figures)	IF with propidium iodide and anti-histone H2 antibody	(62)
2016	Eosinophilic otitis media and Chronic rhinosinusitis	Sinus and ear exudative secretions	Confocal IF with anti-human histone H1 mAb and Hoeschst 33342 DNA staining; SYTOX green	(26)
2017	Chronic obstructive pulmonary disease	Induced sputum plugs	DAPI staining colocalized with anti-MBP antibody and electron microscopy	(25)
2018	Eosinophilic otitis media (case studies)	Bronchial lavage fluid, bronchial secretions and mucus	Confocal IF with anti-human histone H1 mAb and Hoeschst 33342 DNA staining	(48) (63)
2018	Allergic bronchopulmonary aspergillosis	Bronchoscopic mucus plugs	Confocal IF with anti-citrullinated histone H3 mAb and Hoeschst 33342 DNA staining and SEM	(49)

*NB, only in vivo/tissue evidence. in vitro evidence is not given*.

### Delayed resolution of inflammation, inefficient clearance, and autoimmunity

The presence of EETs, as shown in Table 1, has been reported in several inflammatory tissues obtained by biopsy—from bronchial, nasal, skin, esophageal, and intestinal sources, as well as sinus and airway secretions. Against the backdrop of host defense as discussed in the previous section, a plausible outcome of EETs in inflammatory pathology may involve exaggerated responses to microbial stimuli coupled with a reduced capacity in clearance of EET products, such as histones, dsDNA, and peroxidases. Eosinophil extracellular trap (EET) products are all known DAMPs (damage-associated molecular patterns) capable of activating both the innate and adaptive immune systems. Damage-associated molecular patterns (DAMPs) are capable of activating plasmacytoid dendritic cells that present these as self-antigens to cognate lymphocytes, triggering self/autoreactivity (64). For instance, EPX was shown to activate and mobilize dendritic cells to lymph nodes (65). Furthermore, inefficient degradation of DNA-EPX EET products or clearance of EPX released upon ETosis could allow accumulation and subsequent activation of the adaptive system to trigger autoimmune responses. The presence of EETs in tissues with pre-existing autoimmune responses could aggregate immune complexes between autoantibodies and their cognate antigens embedded on ETs. Clearance of such immune complexes by macrophages and dendritic cells could then potentially lead to proinflammatory cytokine secretion, which sustains inflammation (64), interferes with clearance, and prevents resolution, thereby contributing to a vicious cycle of inflammation.

An increased presence of EPX, and FEGs in the airways (sputa) of severe eosinophilic asthmatics were found to be positive predictors of sputum autoantibodies (anti-EPX IgG and anti-nuclear antibodies including anti-dsDNA, anti-histones) (36). Eosinophil extracellular traps (EETs) staining positive for DNA and MBP has been observed in bronchial biopsies obtained from allergic asthmatics that further correlated with infiltrating eosinophils (27). Peripheral blood eosinophils isolated from severe eosinophilic asthmatics compared to non-severe demonstrated higher potential of EET formations *in vitro* when stimulated with LPS and IL-5. The percentage of EET^+^ eosinophils negatively correlated with the lung function. Eosinophil extracellular traps (EETs) were shown to have autocrine effect on inducing further eosinophil degranulation and demonstrated a capability of activating epithelial cells to release pro-inflammatory cytokines (IL-6 and IL-8) (66). In a separate study, EETs were demonstrated to be released on stimulation with thymic stromal lymphopoietin (TSLP), a known Th2 alarmin (61). Thus, in a Th2 environment, EETs could be a potential source of self-antigens. Interestingly, *in vitro* experiments revealed that pharmacologically relevant concentrations of dexamethasone were incapable of reducing EET formation from autoantibody-activated eosinophils (36), hinting at a mechanism for a sustained inflammatory environment in steroid-resistant asthmatics. Furthermore, because of this sustained inflammatory environment, a delayed resolution of EET-induced inflammation may potentially trigger self-reactivity and initiate the production of autoantibodies against ET products such as DNA, histones, and granule proteins, as recently reported in severe asthmatic airways (36).

The concomitant presence of EETs and NETs in COPD sputum was reported recently (25). The authors concluded that EETs may contribute to the severity of COPD. The accumulation of EET-related debris and subsequent phagocytosis by neutrophils may also serve to activate and trigger NETosis, an event that was extensively evident in the high exacerbation severe COPD group (25). It is important to note that in an earlier study, sputum derived from severe COPD patients had detectable anti-nuclear antibodies (ANAs), and an autoimmune pathology for COPD has been proposed (67). However, a direct role of EETs or NETs in the pathogenesis of COPD and any possible underlying autoinflammation has not yet been confirmed. Anti-nuclear antibodies (ANAs) were also reported in nasal exudative secretions from patients with sinus disease and Samster's triad (68), along with evidence of extensive histone-coated EETs in the nasal tissue (26).

Autoantibodies are potent triggers of EETs *in vitro* (36). Sputum with detectable titers of anti-neutrophil cytoplasmic antibody (ANCA), derived from patients diagnosed with eosinophilic granulomatosis and polyangiitis triggered extensive NETs and EETs *in vitro* (69). NETosis and pathogenesis of ANCA is well-known (58), but an avenue has now been opened to decipher the role of EETs in vasculitis and driving pulmonary complications in patients with eosinophilic granulomatosis and polyangiitis. EETs have also been demonstrated in skin biopsies from systemic autoimmune disorders such as Wegner's granulomatosis and bullous pemphigoid (46), as well as intestinal tissue from autoimmune disease such as Crohn's (18) (Table 1).

### Heightened EET response in infection and associated inflammatory pathology

Airway epithelial damage by exaggerated EET anti-bacterial response and collateral tissue damage could possibly explain epithelial (barrier) dysfunction and inflammatory pathology in diseases such as eosinophilic chronic rhinosinusitis (CRS) (70), atopic dermatitis (71), and asthma (72). With respect to intestinal inflammatory diseases, Yousefi et al. (18) demonstrated that increased numbers of eosinophils infiltrated the intestinal lining in *IL-5* transgenic mice after caecal ligation and puncture compared to wild type animals. Increased tissue eosinophilia was associated with an increased level of detectable EETs deposited in the tissue, possibly as a consequence of an exaggerated response to intestinal bacteria. Moreover, the authors showed the presence of EETs in inflamed intestinal tissue from patients with Crohn's disease (18). Again, within eosinophilic asthmatics, there is a subset of patients who suffer from recurrent infective bronchitis (with mixed granulocytic sputum) and low lung function that exhibit autoantibodies in their sputum samples (73). Interestingly, ANAs and anti-EPX IgGs in the sputa of these patients also has the ability to induce EETs *in vitro* (36). It is possible that in addition to NETs, EETs in response to bacterial infection (discussed earlier in section EETs and Bacterial Infection) could be associated with a breakdown of local tolerance as well as a source of potential self-antigens.

### Mucus plugging and sticky secretions

Neutrophil extracellular traps (NETs) have been known to contribute to the viscosity of airway secretions in cystic fibrosis patients (74), and DNase treatment was shown to have clinically relevant improvement (75). Dornase alfa [recombinant human deoxyribonuclease I (rhDNase)] is used therapeutically to cleave the DNA in mucus plugs which improves mucocilliary clearance, and leads to improved pulmonary function. Recent evidence suggests that EETs add substantially to the viscosity of eosinophil-rich exudates of patients with CRS, eosinophilic otitis media and ABPA [reviewed in (35)]. Muniz et al. (49) and Ueki et al. (19) demonstrated the presence of abundant nuclear histone-bearing EETs in mucus secretions obtained from the airways of ABPA patients and nasal secretions of CRS patients, respectively. Compared to NETs, EETs assembled to form more stable aggregates that entrapped fungi and bacteria through hydrophobic interactions, demonstrated *ex vivo* in nasal exudates from CRS patients. When compared to NETs, the EETs exhibited thicker fibers coated by higher number of histone molecules and were less susceptible to proteolytic degradation (19).

Cunha et al. showed that ovalbumin sensitization and challenge in mice led to the accumulation of EET^+^ EPX^+^ live eosinophils in lung tissues and induced increased extracellular DNA in bronchoalveolar lavage (76). In subsequent years, the same group showed that recombinant DNase treatment significantly decreased airway resistance, concomitantly with goblet cell hyperplasia and reduced EET formation (77). Eosinophil extracellular trap (EET) secretions also increased mucin/airway secretions in these mice post-allergen challenge. Recent observations of Dunican et al. (78) showed that mucus plugs in asthmatics correlated with increased airway eosinophilia and EPX content, which occurred in 58% of asthmatics compared to 4.5% of healthy volunteers, and was also associated with reduced lung function. It may be speculated that in asthmatics with increased eosinophilic activity (indicated by increased EPX), there may be a loss of immune tolerance, increasing presence of local autoantibodies, and generation of EETs that contribute to goblet cell hyperplasia and IL-13-related mucin overproduction, both of which lead to the production of highly viscous airway-blocking mucus plugs that are unique to eosinophilic disorders for their exceptionally thick peanut butter-like consistency.

## Conclusions

We now have incontrovertible evidence that EETs form in response to many different stimuli deriving from microbes and their products, as well as non-infectious stimuli such as DAMPs, complement proteins, and immunoglobulins. These traps are important in protection of the host in response to invasive pathogens such as bacteria, and potentially, viruses, fungi, and parasites. However, in the case of chronic, unresolved inflammation, it is apparent that EETs can cause health complications by promoting the production of highly viscous mucus secretions, and potentially setting the stage for the development of autoantibodies. The localized presence of autoantibodies in the lungs (79) is proposed to further exacerbate EET formation, setting up a vicious cycle of inflammation that is not readily ameliorated by glucocorticosteroid treatment. Further studies are anticipated in this area to understand more about how EETs may be prevented in patients with severe inflammatory conditions including steroid-dependent asthma and sinus disease.

## Author contributions

MM prepared the first draft. SU and PL contributed to the development of the manuscript. All authors have read and agreed to the submitted manuscript.

### Conflict of interest statement

The authors declare that the research was conducted in the absence of any commercial or financial relationships that could be construed as a potential conflict of interest.

## Abbreviations

ANA: anti-nuclear antibodies
DAMPs: danger associated molecular patterns
EET: eosinophil extracellular traps
ECP: eosinophil cationic protein
EPX: eosinophil peroxidase
IF: immunofluorescence
MBP: major basic protein
SEM: scanning electron microscopy
LPS: lipopolysaccharide.

## References

[1] Krause JR Boggs DR. Search for eosinopenia in hospitalized patients with normal blood leukocyte concentration. Am J Hematol. (1987) 24:55–63. 10.1002/ajh.2830240108 3799595

[2] Rothenberg ME Hogan SP. THE EOSINOPHIL. Ann Rev Immunol. (2006) 24:147–74. 10.1146/annurev.immunol.24.021605.090720 16551246

[3] Davoine F Lacy P. Eosinophil cytokines, chemokines, and growth factors: emerging roles in immunity. Front Immunol. (2014) 5:570. 10.3389/fimmu.2014.00570 25426119 PMC4225839

[4] Hogan SP Rosenberg HF Moqbel R Phipps S Foster PS Lacy P. Eosinophils: biological properties and role in health and disease. Clin Exp Allergy (2008) 38:709–50. 10.1111/j.1365-2222.2008.02958.x 18384431

[5] Persson C Uller L. Theirs but to die and do: primary lysis of eosinophils and free eosinophil granules in asthma. Am J Resp Crit Care Med. (2014) 189:628–33. 10.1164/rccm.201311-2069OE 24512466

[6] Lacy P Nair P The human eosinophil. In: Wintrobe's Clinical Hematology. 14th ed. Wolters Kluwer, Inc (in press).

[7] Nair P Ochkur SI Protheroe C Radford K Efthimiadis A Lee NA. Eosinophil peroxidase in sputum represents a unique biomarker of airway eosinophilia. Allergy (2013) 68:1177–84. 10.1111/all.12206 23931643 PMC3788081

[8] Acharya KR Ackerman SJ. Eosinophil granule proteins: form and function. J Biol Chem. (2014) 289:17406–15. 10.1074/jbc.R113.546218 24802755 PMC4067173

[9] Lacy P Moqbel R Signalling and degranulation. In: Lee JJ Rosenberg HF, editors. Eosinophils in Health and Disease. San Diego, CA: Academic Press (Elsevier) (2013). p. 206–8.

[10] Scepek S Moqbel R Lindau M. Compound exocytosis and cumulative degranulation by eosinophils and their role in parasite killing. Parasitol Today (1994) 10:276–8. 10.1016/0169-4758(94)90146-5 15275446

[11] Persson C Uller L. Primary lysis of eosinophils as a major mode of activation of eosinophils in human diseased tissues. Nat Rev Immunol. (2013) 13:902. 10.1038/nri3341-c1 24270781

[12] Persson CG Erjefalt JS. “Ultimate activation” of eosinophils *in vivo*: lysis and release of clusters of free eosinophil granules (Cfegs). Thorax (1997) 52:569–74. 10.1136/thx.52.6.569 9227728 PMC1758581

[13] Erjefält J Andersson M Greiff L Korsgren M Gizycki M Jeffery P. Cytolysis and piecemeal degranulation as distinct modes of activation of airway mucosal eosinophils. J Allergy Clin Immunol. (1998) 102:286–94. 9723674 10.1016/s0091-6749(98)70098-3

[14] Erjefält JS Greiff L Andersson M Matsson E Petersen H Linden M. Allergen-induced eosinophil cytolysis is a primary mechanism for granule protein release in human upper airways. Am J Resp Crit Care Med. (1999) 160:304–12. 10390416 10.1164/ajrccm.160.1.9809048

[15] Erjefalt J Greiff L Andersson M Adelroth E Jeffery P Persson C. Degranulation patterns of eosinophil granulocytes as determinants of eosinophil driven disease. Thorax (2001) 56:341–4. 10.1136/thorax.56.5.341 11312400 PMC1746051

[16] Saffari H Hoffman LH Peterson KA Fang JC Leiferman KM Pease LF. Electron microscopy elucidates eosinophil degranulation patterns in patients with eosinophilic esophagitis. J Allergy Clin Immunol. (2014) 133:1728–34.e1. 10.1016/j.jaci.2013.11.024 24439077

[17] Rosenberg HF Foster PS. Reply to eosinophil cytolysis and release of cell-free granules. Nat Rev Immunol. (2013) 13:902. 10.1038/nri3341-c2 24270783

[18] Yousefi S Gold JA Andina N Lee JJ Kelly AM Kozlowski E. Catapult-like release of mitochondrial DNA by eosinophils contributes to antibacterial defense. Nat Med. (2008) 14:949–53. 10.1038/nm.1855 18690244

[19] Ueki S Melo RC Ghiran I Spencer LA Dvorak AM Weller PF. Eosinophil extracellular DNA trap cell death mediates lytic release of free secretion-competent eosinophil granules in humans. Blood (2013) 121:2074–83. 10.1182/blood-2012-05-432088 23303825 PMC3596967

[20] Brinkmann V Reichard U Goosmann C Fauler B Uhlemann Y Weiss DS. Neutrophil extracellular traps kill bacteria. Science (2004) 303:1532–5. 10.1126/science.1092385 15001782

[21] Steinberg BE Grinstein S. Unconventional roles of the NADPH oxidase: signaling, ion homeostasis, and cell death. Science's STKE (2007) 2007:pe11. 10.1126/stke.3792007pe11 17392241

[22] von Köckritz-Blickwede M Goldmann O Thulin P Heinemann K Norrby-Teglund A Rohde M Phagocytosis-independent antimicrobial activity of mast cells by means of extracellular trap formation. Blood (2008) 111:3070–80. 10.1182/blood-2007-07-104018 18182576

[23] Webster SJ Daigneault M Bewley MA Preston JA Marriott HM Walmsley SR. Distinct cell death programs in monocytes regulate innate responses following challenge with common causes of invasive bacterial disease. J Immunol. (2010) 185:2968–79. 10.4049/jimmunol.1000805 20656927 PMC2929480

[24] Mohanan S Horibata S McElwee JL Dannenberg AJ Coonrod SA. Identification of macrophage extracellular trap-like structures in mammary gland adipose tissue: a preliminary study. Front Immunol. (2013) 4:67. 10.3389/fimmu.2013.00067 23508122 PMC3600535

[25] Uribe Echevarría L Leimgruber C García González J Nevado A Álvarez R García LN Evidence of eosinophil extracellular trap cell death in COPD: does it represent the trigger that switches on the disease? Int J Chron Obstruct Pulmon Dis. (2017) 12:885–96. 10.2147/COPD.S115969 28352169 PMC5359000

[26] Ueki S Konno Y Takeda M Moritoki Y Hirokawa M Matsuwaki Y. Eosinophil extracellular trap cell death-derived DNA traps: their presence in secretions and functional attributes. J Allergy Clin Immunol. (2016) 137:258–67. 10.1016/j.jaci.2015.04.041 26070883 PMC4674385

[27] Dworski R Simon HU Hoskins A Yousefi S. Eosinophil and neutrophil extracellular DNA traps in human allergic asthmatic airways. J Allergy Clin Immunol. (2011) 127:1260–6. 10.1016/j.jaci.2010.12.1103 21315435 PMC3085562

[28] Arrieta YR Rojas M Vasquez G Lopez J The lymphocytes stimulation induced DNA release, a phenomenon similar to NETosis. Scand J Immunol. (2017) 86:229–38. 10.1111/sji.12592 28805301

[29] Wartha F Henriques-Normark B. ETosis: a novel cell death pathway. Sci Signal. (2008) 1:pe25. 10.1126/stke.121pe25 18506034

[30] Wen F White GJ VanEtten HD Xiong Z Hawes MC. Extracellular DNA is required for root tip resistance to fungal infection. Plant Physiol. (2009) 151:820–9. 10.1104/pp.109.142067 19700564 PMC2754639

[31] Robb CT Dyrynda EA Gray RD Rossi AG Smith VJ. Invertebrate extracellular phagocyte traps show that chromatin is an ancient defence weapon. Nat Commun. (2014) 5:4627. 10.1038/ncomms5627 25115909 PMC4143918

[32] Muñoz-Caro T Rubio RMC Silva LM Magdowski G Gärtner U McNeilly TN. Leucocyte-derived extracellular trap formation significantly contributes to Haemonchus contortus larval entrapment. Parasit Vectors (2015) 8:607. 10.1186/s13071-015-1219-1 26610335 PMC4661960

[33] Aulik NA Hellenbrand KM Czuprynski CJ. Mannheimia haemolytica and Its leukotoxin cause macrophage extracellular trap formation by bovine macrophages. Infect Immun. (2012) 80:1923–33. 10.1128/IAI.06120-11 22354029 PMC3347434

[34] Yousefi S Simon D Simon HU. Eosinophil extracellular DNA traps: molecular mechanisms and potential roles in disease. Curr Opin Immunol. (2012) 24:736–9. 10.1016/j.coi.2012.08.010 22981682

[35] Ueki S Tokunaga T Fujieda S Honda K Hirokawa M Spencer LA. Eosinophil ETosis and DNA traps: a new look at eosinophilic inflammation. Curr Allergy Asthma Rep. (2016) 16:54. 10.1007/s11882-016-0634-5 27393701 PMC5313036

[36] Mukherjee M Bulir DC Radford K Kjarsgaard M Huang CM Jacobsen EA. Sputum autoantibodies in patients with severe eosinophilic asthma. J Allergy Clin Immunol. (2018) 141:1269–79. 10.1016/j.jaci.2017.06.033 28751233

[37] Prince LR Graham KJ Connolly J Anwar S Ridley R Sabroe I. *Staphylococcus aureus* induces eosinophil cell death mediated by α-hemolysin. PLoS ONE (2012) 7:e31506. 10.1371/journal.pone.0031506 22355374 PMC3280314

[38] Weiler CR Kita H Hukee M Gleich GJ. Eosinophil viability during immunoglobulin-induced degranulation. J Leukoc Biol. (1996) 60:493–501. 10.1002/jlb.60.4.493 8864134

[39] Fukuda T Ackerman SJ Reed CE Peters MS Dunnette SL Gleich GJ. Calcium ionophore A23187 calcium-dependent cytolytic degranulation in human eosinophils. J Immunol. (1985) 135:1349–56. 3925008

[40] Gevaert E Yousefi S Bachert C Simon HU. Reply. J Allergy Clin Immunol. (2018) 141:1164–5. 10.1016/j.jaci.2017.05.048 28780969

[41] Gevaert E Zhang N Krysko O Lan F Holtappels G De Ruyck N. Extracellular eosinophilic traps in association with Staphylococcus aureus at the site of epithelial barrier defects in patients with severe airway inflammation. J Allergy Clin Immunol. (2017) 139:1849–60.e1846. 10.1016/j.jaci.2017.01.019 28216437

[42] Ueki S Tokunaga T Melo RCN Saito H Honda K Fukuchi M. Charcot-Leyden crystal formation is closely associated with eosinophil extracellular trap cell death. Blood (2018). 132, 2183–2187. 10.1182/blood-2018-04-842260 30154112 PMC6238188

[43] Minai-Fleminger Y Gangwar RS Migalovich-Sheikhet H Seaf M Leibovici V Hollander N. The CD48 receptor mediates *Staphylococcus aureus* human and murine eosinophil activation. Clin Exp Allergy (2014) 44:1335–46. 10.1111/cea.12422 25255823

[44] Peachman KK Lyles DS Bass DA Mitochondria in eosinophils: functional role in apoptosis but not respiration. Proc Natl Acad Sci USA. (2001) 98:1717–22. 10.1073/pnas.98.4.1717 11172017 PMC29323

[45] McDonald B Urrutia R Yipp Bryan G Jenne Craig N Kubes P. Intravascular neutrophil extracellular traps capture bacteria from the bloodstream during sepsis. Cell Host Microbe (2012) 12:324–33. 10.1016/j.chom.2012.06.011 22980329

[46] Simon D Hoesli S Roth N Staedler S Yousefi S Simon HU. Eosinophil extracellular DNA traps in skin diseases. J Allergy Clin Immunol. (2011) 127:194–9. 10.1016/j.jaci.2010.11.002 21211654

[47] Zureik M Neukirch C Leynaert B Liard R Bousquet J Neukirch F. Sensitisation to airborne moulds and severity of asthma: cross sectional study from European Community respiratory health survey. BMJ (2002) 325:411. 10.1136/bmj.325.7361.411 12193354 PMC119432

[48] Omokawa A Ueki S Kikuchi Y Takeda M Asano M Sato K. Mucus plugging in allergic bronchopulmonary aspergillosis: implication of the eosinophil DNA traps. Allergol Int. (2018) 67:280–2. 10.1016/j.alit.2017.08.002 28886913

[49] Muniz VS Silva JC Braga YAV Melo RCN Ueki S Takeda M. Eosinophils release extracellular DNA traps in response to *Aspergillus fumigatus*. J Allergy Clin Immunol. (2018) 141:571–85.e7. 10.1016/j.jaci.2017.07.048 28943470

[50] Bruns S Kniemeyer O Hasenberg M Aimanianda V Nietzsche S Thywißen A. Production of extracellular traps against aspergillus fumigatus *in vitro* and in infected lung tissue is dependent on invading neutrophils and influenced by hydrophobin RodA. PLoS Pathog. (2010) 6:e1000873. 10.1371/journal.ppat.1000873 20442864 PMC2861696

[51] Gazendam R Hamme JV Tool A Hoogenboezem M Berg JVD Prins J. Human neutrophils use different mechanisms to kill *Aspergillus fumigatus* conidia and hyphae: evidence from phagocyte defects. J Immunol. (2016) 196:1272–83. 10.4049/jimmunol.1501811 26718340

[52] Ashar HK Mueller NC Rudd JM Snider TA Achanta M Prasanthi M. The role of extracellular histones in influenza virus pathogenesis. Am J Pathol. (2018) 188:135–48. 10.1016/j.ajpath.2017.09.014 29107075 PMC5745522

[53] Souza PSS Barbosa LV Diniz LFA da Silva GS Lopes BRP Souza PMR. Neutrophil extracellular traps possess anti-human respiratory syncytial virus activity: Possible interaction with the viral F protein. Virus Res. (2018) 251:68–77. 10.1016/j.virusres.2018.04.001 29621602

[54] Rosenberg HF Dyer KD Domachowske JB. Respiratory viruses and eosinophils: exploring the connections. Antiviral Res. (2009) 83:1–9. 10.1016/j.antiviral.2009.04.005 19375458 PMC2741084

[55] Percopo CM Dyer KD Ochkur SI Luo JL Fischer ER Lee JJ. Activated mouse eosinophils protect against lethal respiratory virus infection. Blood (2014) 123:743–52. 10.1182/blood-2013-05-502443 24297871 PMC3907759

[56] Kaplan MJ Radic M Neutrophil extracellular traps (NETs): double-edged swords of innate immunity. J Immunol. (2012) 189:2689–95. 10.4049/jimmunol.1201719 22956760 PMC3439169

[57] Panda R Krieger T Hopf L Renné T Haag F Röber N. Neutrophil extracellular traps contain selected antigens of anti-neutrophil cytoplasmic antibodies. Front Immunol. (2017) 8:439. 10.3389/fimmu.2017.00439 28450870 PMC5389973

[58] Jennette JC Falk RJ Hu P Xiao H. Pathogenesis of antineutrophil cytoplasmic autoantibody–associated small-vessel vasculitis. Ann Rev Pathol. (2013) 8:139–60. 10.1146/annurev-pathol-011811-132453 23347350 PMC5507606

[59] Corsiero E Pratesi F Prediletto E Bombardieri M Migliorini P. NETosis as source of autoantigens in rheumatoid arthritis. Front Immunol. (2016) 7:485. 10.3389/fimmu.2016.00485 27895639 PMC5108063

[60] Persson C Ueki S. Lytic eosinophils produce extracellular DNA traps as well as free eosinophil granules. J Allergy Clin Immunol. (2018) 141:1164. 10.1016/j.jaci.2017.05.047 28780970

[61] Simon D Radonjic-Hosli S Straumann A Yousefi S Simon HU. Active eosinophilic esophagitis is characterized by epithelial barrier defects and eosinophil extracellular trap formation. Allergy (2015) 70:443–52. 10.1111/all.12570 25620273

[62] Wouters J Waelkens E Vandoninck S Segaert S van den Oord JJ. Mass spectrometry of flame figures. Acta Derm Venereol. (2015) 95:734–5. 10.2340/00015555-2050 25613159

[63] Ohta N Ueki S Konno Y Hirokawa M Kubota T Tomioka-Matsutani S. ETosis-derived DNA trap production in middle ear effusion is a common feature of eosinophilic otitis media. Allergol Int. (2018) 67:414–16. 10.1016/j.alit.2017.11.007 29242145

[64] Mbongue J Nicholas D Firek A Langridge W. The role of dendritic cells in tissue-specific autoimmunity. J Immunol Res. (2014) 2014:857143. 10.1155/2014/857143 24877157 PMC4022068

[65] Chu DK Jimenez-Saiz R Verschoor CP Walker TD Goncharova S Llop-Guevara A. Indigenous enteric eosinophils control DCs to initiate a primary Th2 immune response *in vivo*. J Exp Med. (2014) 211:1657–72. 10.1084/jem.20131800 25071163 PMC4113937

[66] Choi Y Le Pham D Lee DH Lee SH Kim SH Park HS. Biological function of eosinophil extracellular traps in patients with severe eosinophilic asthma. Exp Mol Med. (2018) 50:104. 10.1038/s12276-018-0136-8 30115903 PMC6095846

[67] Morissette M Jobse B Thayaparan D Nikota J Shen P Labiris N. Persistence of pulmonary tertiary lymphoid tissues and anti-nuclear antibodies following cessation of cigarette smoke exposure. Resp Res. (2014) 15:49. 10.1186/1465-9921-15-49 24754996 PMC4021094

[68] Tan BK Li QZ Suh L Kato A Conley DB Chandra RK. Evidence for intranasal antinuclear autoantibodies in patients with chronic rhinosinusitis with nasal polyps. J Allergy Clin Immunol. (2011) 128:1198–206.e1. 10.1016/j.jaci.2011.08.037 21996343 PMC3384688

[69] Mukherjee M Thomas SR Radford K Dvorkin-Gheva A Davychenko S Kjarsgaard M. Sputum ANCA in serum ANCA-negative eosinophilic granulomatosis with polyangiitis (eGPA). Am J Respir Crit Care Med. (2018). 10.1164/rccm.201804-0809OC. [Epub ahead of print]. 30179583

[70] Tieu DD Kern RC Schleimer RP. Alterations in epithelial barrier function and host defense responses in chronic rhinosinusitis. J Allergy Clin Immunol. (2009) 124:37–42. 10.1016/j.jaci.2009.04.045 19560577 PMC2802265

[71] Kim BE Leung DYM. Significance of skin barrier dysfunction in atopic dermatitis. Allergy Asthma Immunol Res. (2018) 10:207–15. 10.4168/aair.2018.10.3.207 29676067 PMC5911439

[72] Gon Y Hashimoto S. Role of airway epithelial barrier dysfunction in pathogenesis of asthma. Allergol Int. (2018) 67:12–17. 10.1016/j.alit.2017.08.011 28941636

[73] Chu DK Al-Garawi A Llop-Guevara A Pillai RA Radford K Shen P. Therapeutic potential of anti-IL-6 therapies for granulocytic airway inflammation in asthma. Allergy Asthma Clin Immunol. (2015) 11:1–6. 10.1186/s13223-015-0081-1 25878673 PMC4397814

[74] Papayannopoulos V Staab D Zychlinsky A. Neutrophil elastase enhances sputum solubilization in cystic fibrosis patients receiving DNase therapy. PLoS ONE (2011) 6:e28526. 10.1371/journal.pone.0028526 22174830 PMC3235130

[75] VanDevanter Donald R Craib Marcia L Pasta David J Millar Stefanie J Morgan Wayne J Konstan Michael W. Cystic fibrosis clinical characteristics associated with dornase alfa treatment regimen change. Pediatr Pulmonol. (2017) 53:43–9. 10.1002/ppul.23897 29064184

[76] Cunha AA Porto BN Nuñez NK Souza RG Vargas MH Silveira JS. Extracellular DNA traps in bronchoalveolar fluid from a murine eosinophilic pulmonary response. Allergy (2014) 69:1696–700. 10.1111/all.12507 25130372

[77] da Cunha AA Nuñez NK de Souza RG Moraes Vargas MH Silveira JS Antunes GL. Recombinant human deoxyribonuclease therapy improves airway resistance and reduces DNA extracellular traps in a murine acute asthma model. Exp Lung Res. (2016) 42:66–74. 10.3109/01902148.2016.1143537 27070484

[78] Dunican EM Elicker BM Gierada DS Nagle SK Schiebler ML Newell JD. Mucus plugs in patients with asthma linked to eosinophilia and airflow obstruction. J Clin Investig. (2018) 128:997–1009. 10.1172/JCI95693 29400693 PMC5824874

[79] Mukherjee M Nair P. Autoimmune responses in severe asthma. Allergy Asthma Immunol Res. (2018) 10:428–47. 10.4168/aair.2018.10.5.428 30088364 PMC6082822

